# Determining the resolution of a tracer for magnetic particle imaging by means of magnetic particle spectroscopy

**DOI:** 10.1039/d3ra01394d

**Published:** 2023-05-24

**Authors:** Amani Remmo, Frank Wiekhorst, Olaf Kosch, Stefan Lyer, Harald Unterweger, Harald Kratz, Norbert Löwa

**Affiliations:** a Physikalisch-Technische Bundesanstalt Berlin, Metrology for Magnetic Nanoparticles Abbestr. 2-12 10587 Berlin Germany amani.remmo@ptb.de; b Department of Otorhinolaryngology, Head and Neck Surgery, Section of Experimental Oncology and Nanomedicine (SEON), Professorship for AI-Controlled Nanomaterials, Universitätsklinikum Erlangen Erlangen Germany; c Charité-Universitätsmedizin Berlin, Clinic for Radiology Charitéplatz 1 10117 Berlin Germany

## Abstract

Magnetic particle imaging (MPI) is an imaging modality to quantitatively determine the three-dimensional distribution of magnetic nanoparticles (MNPs) administered as a tracer into a biological system. Magnetic particle spectroscopy (MPS) is the zero-dimensional MPI counterpart without spatial coding but with much higher sensitivity. Generally, MPS is employed to qualitatively evaluate the MPI capability of tracer systems from the measured specific harmonic spectra. Here, we investigated the correlation of three characteristic MPS parameters with the achievable MPI resolution from a recently introduced procedure based on a two-voxel-analysis of data taken from the system function acquisition that is mandatory in Lissajous scanning MPI. We evaluated nine different tracer systems and determined their MPI capability and resolution from MPS measurements and compared the results with MPI phantom measurements.

## Abstract 2

We evaluated 9 tracers by magnetic particle spectroscopy to estimate their magnetic particle imaging capability and investigated the correlation of 3 MPS parameters and the hydrodynamic size distribution with the achievable MPI resolution *r* determined by two-voxel-analysis.

## Graphical abstract

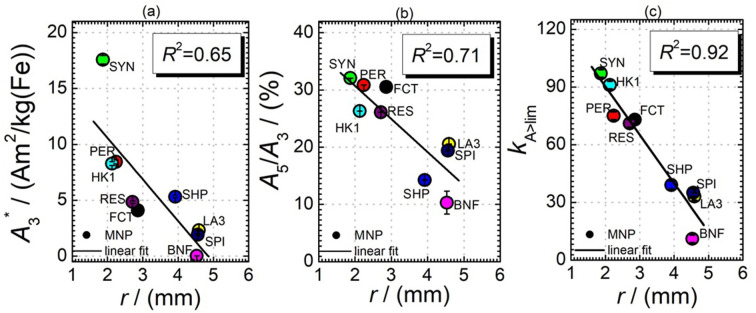

## 1. Introduction

Magnetic particle imaging (MPI) is a quantitative three-dimensional imaging technique capable of specifically detecting magnetic nanoparticles (MNPs) administered as a tracer into a biological system.^1–3^ MPI offers several advantages over current clinically used imaging techniques, such as zero signal attenuation by tissue, radiation-free tracer materials, and high spatial and temporal resolution.^1,4^ Since its first presentation in 2005 (ref. 1 and 5) MPI has made considerable technical progress in recent years making commercial MPI scanners presently available for preclinical investigations of animal models up to rat size.^2^ Only tracers with very specific properties exhibit a sufficiently high MPI signal.^1,6^ The properties of the tracers depend on the biological environment and the binding state,^7^ if they relax by Brownian relaxation. Thus, to successfully conduct experiments, performance measurements for MPI must take place in advance.

There are two main MPI reconstruction techniques: *f*-space and *x*-space.^8,9^ The *x*-space MPI (magnetic insight) is simpler but requires the exact location of the field-free point at each time point for image generation.^8^ In contrast, the *f*-space MPI (Bruker) needs the complex calibration and reconstruction of the tracer distribution. To evaluate the MPI performance of tracers, *x*-space MPI (where no inversion step or deconvolution is required for image generation) uses MPS devices that record a point-spread function of which peak width and height are used to estimate how much a given MNP distribution appears spatially enlarged (“smeared into neighbouring voxels”).^8^ However, this does not include the benefit of an inversion step or deconvolution within *x*-space or *f*-space MPI. By the inversion step of *f*-space MPI the smearing is reduced.^10^ However, it makes the result more sensitive to noise.

**Fig. 1:**
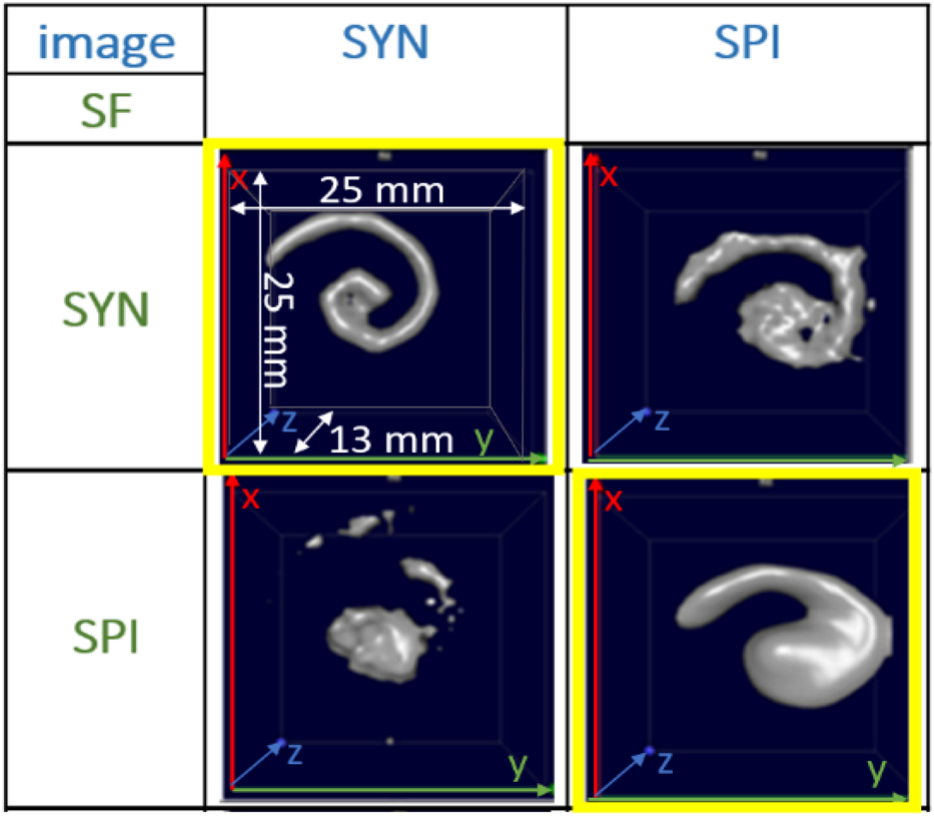
MPI image reconstructions of the resolution (spiral) phantom using different tracer – SF combinations. Two tracer types SYN (left column, Synomag-D, details of the tracers are summarized in Table 1, see Section 2.1) and SPI (right column, SPION-citrate) have been measured in water (top row) in the spiral phantom. The temperature was kept constant at 20 °C during the measurements and the positioning of the phantoms filled with liquid tracer was done using the animal support (rat bed). The position varied by several millimeters. The reconstructions were carried out with each of the two SFs that were acquired using a reference sample of 4 μL at an iron concentration *c*(Fe) = 45 mmol L^−1^ (total iron amount of 10 μg) over a regular grid of 25 × 25 × 13 voxels (single voxel size 1 mm^3^), so that one SF recording took about 9 h.

For the *f*-space MPI, there are several methods to evaluate the MPI performance of tracers. One is to use, resolution phantoms, see Fig. 1. A prerequisite for the reconstruction of the tracer distribution in resolution phantoms is the time-consuming acquisition (typically one day acquisition time) of a so-called system function (SF). This type of calibration is mandatory for the MPI reconstruction since each tracer system has its own specific magnetic response when exposed to the AC excitation fields.^8,11^ To this end, the SF contains numerous calibration scans that are performed using a point-like reference sample of the tracer type intended for the investigation, which is positioned by a robot at a series of defined positions within the field-of-view (FoV). The impact of the tracer type on the MPI image quality can be seen in Fig. 1 on the yellow marked diagonal elements (tracer type is identical for phantom measurement and SF acquisition). Each of the two different iron oxide multi-core tracers possesses an individual MPI tracer performance. Where the spiral filled with Synomag-D can be resolved very precisely (upper left panel of Fig. 1), the spiral containing SPION-citrate tracer (central panel) exhibits a slightly reduced resolution. The importance of the correct SF is displayed at the off-diagonal panels of Fig. 1. Here, a SF of a different tracer type at the same iron concentration has been used for the image reconstruction, deliberately. In all cases, the image quality is heavily distorted so that nearly no geometry details of the spiral are resolved.

This demonstrates that the MPI image quality is crucially depending on the tracer type and that the selection of a suitable SF for image reconstruction plays an important role for the resolution and quality of MPI images.

Another, much more effective way to assess the tracer performance can be achieved by using magnetic particle spectrometers (MPS), *i.e.*, detecting the dynamic non-linear magnetic susceptibility waiving any spatial resolution. These technically simpler MPS devices are easier and faster (measurement takes a few seconds) to operate, less expensive and more sensitive.^3^ Usually the MPS parameters 
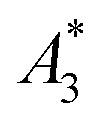
 and *A*_5_/*A*_3_ are used to evaluate the tracer performance.^12^ On the one hand, such simplified considerations of parameters do not give any information about Néel or Brownian relaxation of the parameters under given measurement parameters. Néel and Brownian signal contributions depends on both particle and measurement parameters.^13,14^ Especially, the Brownian contribution generally will be impacted by the presence of binding states (changing the mobility of the particles). On the other hand, usually a wideband harmonic response should be incorporated to evaluate the tracer performance. 
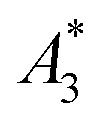
 and *A*_5_/*A*_3_ just give a first hint but the higher harmonics (required for *f*-space reconstruction) are even more important – especially in case that you are interested in image details. Taking only the lower harmonics (such as *A*_3_ and *A*_5_) would result in a blurred blob in the reconstructed image in cases where higher harmonics are not present. Here we additionally considered the number of harmonics *A*_*k*_ with amplitudes above the limit of detection (LOD) *k*_*A*>LOD_ of the MPS device to evaluate the MPS signal in relation to the noise and to consider the dependence of the inversion step on the noise. The duration of the SF acquisition depends on the repetition time that is different for 2D or 3D imaging, on the use of focus fields to move (enlarge) the FOV, and on the number of averages. Since the acquisition of a SF for a single tracer system takes about 9 h (see Fig. 1), prior prediction of tracer behaviour in MPI using MPS would be beneficial.

Besides magnetic parameters obtained by MPS, physical parameters such as the total (hydrodynamic) size and size distribution have been correlated with MPI performance.^6,15^ These parameters are often relevant in specific MPI applications, *e.g.*, for imaging of vessel flows where small and stable MNP are preferable.^16–18^ In addition, the total size of a tracer becomes relevant when it is changing due to aggregate formation.^17,19^ The thereby increased magnetic (dipole–dipole) interactions between the particles in an aggregate lead to a strong decrease of the signal from a tracer.^19^

In this work we investigated the capability of MPS to obtain reliable, quantitative values for resolution and detection limits of a tracer system without the need of time-consuming MPI acquisition of SFs. For nine different tracer systems, we first correlated characteristic MPS parameters as well as the hydrodynamic diameter and size distribution extracted from dynamic light scattering (DLS) to the resolution *r* obtained by the recently introduced two-voxel-analysis of MPI SFs.^20^ To demonstrate the performance of our analysis we compared the results with phantom measurements of a spiral phantom developed for MPI resolution measurements.^21^

## 2. Experimental

This chapter summarizes the experimental methods and materials that have been used in this work.

### 2.1 Magnetic nanoparticle systems and media

We used nine different tracers providing a broad range of magnetic properties. Details of origin, coating, and some physical parameters of the tracer are summarized in Table 1. All chemicals used in this study were of analytical reagent grade. Deionized water (ddH_2_O) was used as dilution medium, *e.g.*, to adjust the tracer concentration.

**Table 1:** Summary of the nine different tracer systems used in this work^a^

Sample name	ID	Product information	MPS			MPI	DLS	
			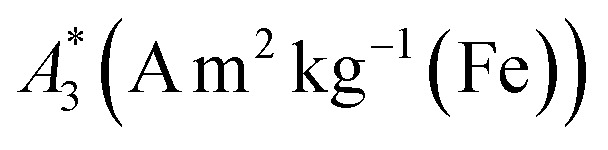	*A*_5_/*A*_3_ (%)	*k*_*A*>LOD_	*r* (mm)	*d*_hyd_ (nm)	PDI
Ferucarbotran	FER	Meito Sangyo	4.1(1)	30.525(3)	73	2.8	59.42(2)	0.2(4)
		LOT: DDM128N/S1-007 surface: carboxy dextran						
Perimag	PER	Micromod Partikeltechnologie GmbH	8.4(2)	30.862(2)	75	2.24	139.4(1)	0.22(1)
		LOT: 05216102-02 surface: plain						
Synomag	SYN	Micromod Partikeltechnologie GmbH	17.6(4)	32.073(2)	97	1.87	45.8(7)	0.05(2)
		LOT: 01517104-04 surface: dextran-plain						
SHP30-10	SHP	Ocean Nanotech	5.3(1)	14.25(3)	39	3.92	70.5(7)	0.14(1)
		LOT: 17318SHP surface: carboxylic acid						
HK111	HK1	Charité-Experimental Radiology	8.3(2)	26.325(5)	91	2.13	57.6(2)	0.12(3)
		LOT: HK2015 surface: carboxy methyl dextran						
BNF-Dextran	BNF	Micromod Partikeltechnologie GmbH	0.0344(7)	10(2)	11	4.53	130.3(5)	0.13(5)
		LOT: 1131684-01 surface: dextran						
La32-BSA	LA3	Universitätsklinikum Erlangen	2.32(5)	20.55(3)	33	4.59	75(1)	0.17(1)
		LOT: SEONLA32-BSA1 surface: Lauric acid						
Spion citrat	SPI	Universitätsklinikum Erlangen	1.93(4)	19.39(4)	35	4.56	50.3(9)	0.28(1)
		LOT: SEONC16 surface: citrate						
Resovist®	RES	Bayer HealthCare	4.8(1)	26.096(9)	71	2.71	58.3(1)	0.29(5)
		LOT: 21 016 surface: carboxy dextran						

a Given are the sample name, the ID used as abbreviation in graphs and text together with the name of the supplier and the coating of the tracer. Furthermore, the hydrodynamic diameter *d*_hyd_ (*z*-average), polydispersity index (PDI) obtained by DLS and characteristic MPS parameters 
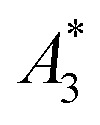
 (*A*_3_) normalized to iron amount and shape parameter *A*_5_/*A*_3_ at *f*_0_ = 25 kHz at an excitation field *B*_ex_ = 12 mT as determined for the stock suspension) are presented. The MPS measurements were performed at *B* = 12 mT. The limit of detection for the iron amount (determined from the amplitude LOD (*A*_3_) = 3 × 10^−12^ A m^2^) was determined as the iron mass of a tracer corresponding, where LOD (*A*_3_) was defined as three times the standard deviation of *A*_3_ resulting from 10 blank measurements after correction for the empty sample background. The uncertainty for *k*_*A*>LOD_ was estimated geometrically as ±2. The numbers in parentheses denote the uncertainty of the last digit, *e.g.*, 58.3(1) reads as 58.3 ± 0.3 nm.

### 2.2 Dynamic light scattering (DLS)

Dynamic light scattering (DLS) was used to determine the hydrodynamic diameter (*d*_hyd_) and the size distribution (PDI-polydispersity index) of the tracers. We used the Zetasizer Nano-ZS (Malvern U.K.) equipped with a green laser (wavelength 633 nm). All measurements were performed at 21 °C. A sample volume of 400 μL was transferred in a square 10 × 10 mm disposable polystyrene cuvette at an iron concentration *c*(Fe) of 5 mmol L^−1^. The experiments were conducted in backscattering mode at a scattering angle of 173°. Assuming spherical noninteracting particles, *d*_hyd_ was obtained from the diffusion coefficient using the Stokes–Einstein relation.^22^

### 2.3 Magnetic particle spectroscopy (MPS)

MPS measurements were performed using a commercial device (MPS-3, Bruker, Germany) operating at an amplitude *B*_ex_ = 12 mT and a frequency *f*_0_ = 25 kHz. MPS detects the non-linear dynamic magnetic response of tracer exposed to an alternating magnetic field. For the measurement a fast reaction tube (Applied Biosystems®, MicroAmp) containing a sample volume of *V* = 10 μL and an iron concentration of *c*(Fe) = 45 mmol L^−1^ is placed into the (gradiometeric) detection coil of the MPS detecting the induced dynamic magnetization. By Fourier transformation of the time signal the spectral components are obtained showing distinctive amplitudes at odd multiples (harmonics) of the excitation frequency *f*_0_. We considered three characteristic parameters of the MPS harmonic spectra, the amplitude of the third harmonic normalized to the iron amount of the sample 
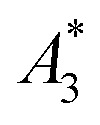
, the (concentration independent) ratio between 5th and 3rd harmonic *A*_5_/*A*_3_, and the number of MPS harmonics *A*_*k*_ above the limit of detection (LOD) *k*_*A*>LOD_ of the MPS device.

The limit of detection (LOD) of the MPS was determined according to the guidance of the International Union of Pure and Applied Chemistry (IUPAC): LOD (*A*_*k*_) = *μ* + 3*σ* of ten empty-sample-holder (background) measurements. For 
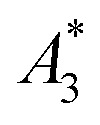
, the uncertainty was performed with the uncertainty contributions of the iron concentration *c*, the amplitude *A*_3_, and the volume *V*.^23^ The relative uncertainty for *c* and *V* was assumed to be 1.5%. A relative uncertainty of 1.5% was assumed for *c* and *V*. For the concentration independent *A*_5_/*A*_3_, only the noise contributions were considered. The MPS noise of *A*_5_ and *A*_3_ was determined using 20 blank measurements by calculating the standard deviation from the 20 individual measurements. The uncertainty for *k*_*A*>LOD_ was estimated geometrically as ±2.

### 2.4 Magnetic particle imaging (MPI)

MPI measurements were performed with a commercial, preclinical MPI scanner (Bruker MPI 25/50 FF) based on the field-free point (FFP) approach. The FFP is generated by a static magnetic field gradient (1.25/1.25/2.5 T m^−1^ in *x*/*y*/*z*-direction).^1,5,24^ By superimposing oscillating magnetic drive fields with field amplitudes of 12/12/12 mT and frequencies 24.5/26.0/25.3 kHz the FFP is moved on a Lissajous trajectory.^25^ Gradiometeric receive coils measured the time signal from which the harmonic spectrum was derived by Fourier transformation.^26^ The spectral pattern of the MPI signal (*ū*) depends on the tracer system, the environmental conditions and the spatial distribution of the tracer within the FoV. A three-dimensional image reconstruction of the tracer distribution (*c*) is performed by solving the following least-squares problem using the Kaczmarz algorithm with Tikhonov regularization (regularization parameter *λ*):‖*S̄c* − *ū*‖^2^ + *λ*‖*c*‖^2^ → min.^27^

#### 2.4.1 System function (SF)

The SF (*S̄*) acquisition can be considered as a calibration procedure measuring the MPI signal *ū* of a point-like tracer sample at each spatial position of the FoV in the MPI scanner. The SFs were recorded using 100 averages subtracted by a background measurement. The SF for the nine tracers has been recorded in a volume of 25 × 25 × 13 voxel, with a voxel size of 1 × 1 × 1 mm^3^. The SF acquisition took 9 h. The volume of the point-like sample was *V*_ref_ = 4 μL (2 × 2 × 1 mm^3^) with an iron concentration of 45 mmol L^−1^. The same settings as for the SFs were used for an empty scanner measurement and for measurements of the resolution phantoms described in ref. 21.

#### 2.4.2 Two-voxel-analysis and resolution (spiral) phantom

The two-voxel-analysis is an efficient procedure to determine the achievable resolution in MPI directly from a SF measurement to synthetically construct MPI measurements of two point-like tracer samples at varying distances.^20^ To simulate an array of two different tracers sources, the corresponding data are extracted from the SF and superimposed, with the additional inclusion of noise data from an empty scanner measurement, giving the total complex voltage signal *ū*. We only used frequency components of the SF above a certain signal-to-noise (SNR > 4) level, otherwise the reconstruction of the tracer distribution would be based on the noise of the SF acquisition. By inversion of *S̄c* = *ū*, the resulting tracer distribution *c* is reconstructed for the case of two voxels separated by one empty voxel. The result is normalized to the iron content of the voxel.^20^ The image resolution *r* is then determined by the distance at which the minimum has decreased to 50% of the maximum tracer content. To compare the resolution of different tracer systems, we used a spiral resolution phantom as introduced in ref. 21.

## 3. Results and discussion

From the recorded MPS signals of the MPI tracer selection we extracted the specific amplitude 
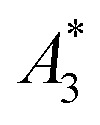
, the concentration independent *A*_5_/*A*_3_ value as well as the number of harmonics above the LOD *k*_*A*>LOD_ (see Table 1).

The 
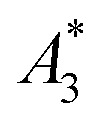
 of the investigated tracers varied between 
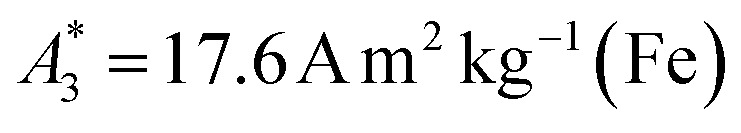
 for SYN and 
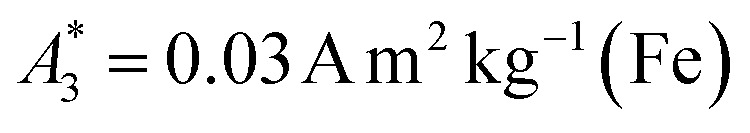
 for BNF. Note, that 
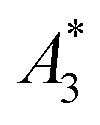
 of the MPI gold standard RES was 4.8 A m^2^ kg^−1^ (Fe). This demonstrates the broad variation of the dynamic responses of potential MPI tracer systems and the excellent sensitivity and huge dynamic range (about five orders of magnitude) of the MPS device.

Only information-carrying harmonics above the noise level should be used for image reconstruction in MPI. Therefore, it is generally expected that the flatter the decay of the amplitudes *A*_*k*_ in the measured MPS spectrum of a tracer, the better the spatial resolution after MPI image reconstruction. The slope of the odd harmonics (in MPS spectra even harmonic have zero amplitude due to the symmetry of the magnetization curve and in the absence of any offset field) is parametrized by the *A*_5_/*A*_3_ value as a first order estimation. Although for high MPI resolution in *f*-space MPI, a wideband harmonic response is mandatory, a high *A*_5_/*A*_3_ value is often associated with high resolution in MPI. MPI spectra contain even and odd harmonics and more complex patterns due to the three slightly different excitation frequencies and the gradient (offset) fields used for spatial encoding (see 2.4). Corresponding to the 
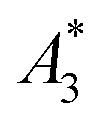
 value, the *A*_5_/*A*_3_ value of the tested tracers also varied between *A*_5_/*A*_3_ = 32.1% for SYN and *A*_5_/*A*_3_ = 10% for BNF. The *A*_5_/*A*_3_ of the well known RES sample was 26.1%.

**Fig. 2:**
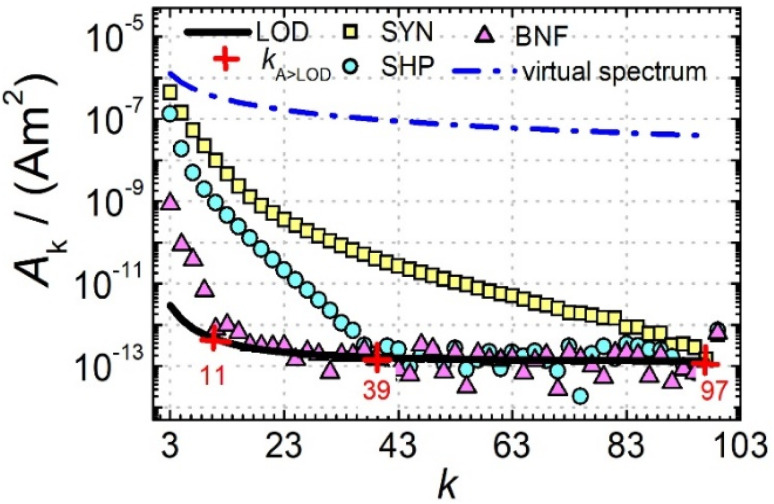
MPS spectra of the tracers SYN, SHP, and BNF and the virtual spectrum of a tracer system with step-functional magnetization behavior and an *M*_s_ of 120 A m^2^ kg^−1^ (Fe) (blue line). Furthermore, the mean noise floor of ten background measurements (black line) is indicated to determine the number *k*_*A*>LOD_ (red crosses) of harmonics *A*_*k*_. All tracers were measured at *B*_ex_ = 12 mT, with a volume of 10 μL at an iron concentration of *c*(Fe) = 45 mmol L^−1^.

Next, we further analysed the number of amplitudes *A*_*k*_ above the limit of detection (LOD) *k*_*A*>LOD_ of the MPS system as another parameter to describe the MPI performance of a tracer. Fig. 2 shows MPS spectra at *B*_ex_ = 12 mT for the three tracers SYN, SHP and BNF (obtained at an iron concentration *c*(Fe) = 45 mmol L^−1^) together with the LOD (black line) for each harmonic starting at about 3 × 10^−12^ A m^2^ that was determined as the mean +3 times the standard deviation of ten background measurements at the same excitation field.

An “ideal” reaction of a tracer exposed to the oscillating magnetic excitation fields used in MPI or MPS would be the immediate switching of the magnetization towards its saturation value *M*_S_ even at the smallest field amplitude, which is mathematically described by a step function centered at the origin *B* = 0 with an amplitude ±*M*_S_.^28^

For a step function, the harmonics *A*_*k*_ of the Fourier transform can be expressed analytically by

1 *A*_*k*_ = 4*M*_S_/(π*k*)

for odd *k* = 1, 3, 5, 7, … and 0 for even *k*.^29^ Therefore, the highest possible *A*_3_ would be given as *A*_3_ = 4/(3π)*M*_S_ ∼0.42*M*_S_. Assuming a saturation magnetization of about 120 A m^2^ kg^−1^ (Fe) often reported for magnetite or maghemite MNP,^30^ this means that for tracers based on iron oxide a maximum value of about 
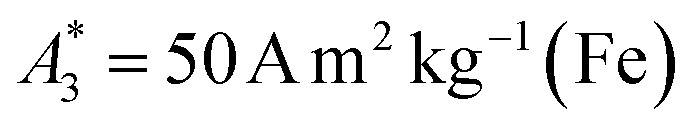
 can be achieved. In addition to the MPS spectra *A*_*k*_ of the three tracers, Fig. 2 show the *A*_*k*_ of the step function curve with the *M*_s_ of 120 A m^2^ kg^−1^ (Fe) (blue line) at an iron concentration of 45 mmol L^−1^. This theoretical curve represents the MPS spectrum of iron oxide particles without phase shift, therefore there are no tracers that will exhibit higher *A*_*k*_ than this MPS spectrum. In a same way, the maximum *A*_5_/*A*_3_ that can be reached is 4*M*_S_/(5π)/(4*M*_S_/(3π)) = 3/5 = 0.6 and therefore no better (flatter) *A*_5_/*A*_3_ than 60% can be obtained. As can be seen, *k*_*A*>LOD_ strongly varies for the three tracer types. SYN produces a rich harmonic spectrum with 97 harmonics above the LOD whereas for BNF only 11 harmonics can be detected with the MPS device, which is the highest and lowest value of all investigated tracers, respectively. For RES 71 harmonics can be detected with MPS.

**Fig. 3:**
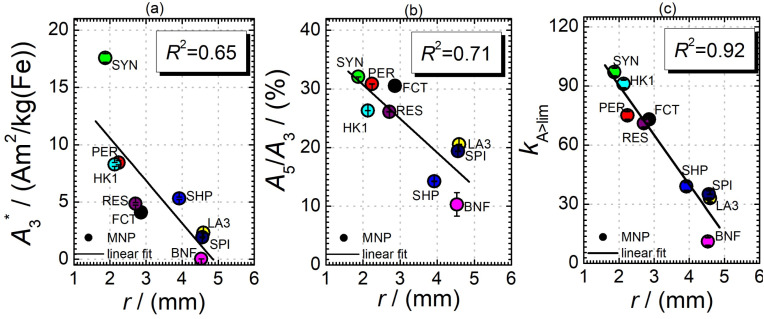
Specific MPS amplitude 
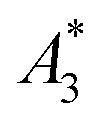
 (a), harmonic ratio *A*_5_/*A*_3_ (b), and number of harmonics above LOD *k*_*A*>LOD_ (c) determined from MPS measurements as a function of the resolution *r* obtained by the two-voxel-analysis from MPI.

Next, we evaluated the relation between MPI resolution *r* obtained from the two-voxel-analysis for each tracer and the three characteristic MPS parameters (
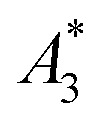
, *A*_5_/*A*_3_ and *k*_*A*>LOD_), see Fig. 3. For all tracer systems a resolution *r* in the range 1.5 mm to 5 mm was found with SYN exhibiting the best resolution *r* = 1.9 mm. The determining factor for the best achievable resolution *r* is mainly the number of frequency components (FC) of signal *ū* above the noise floor that can be used in the reconstruction. The number and the strength especially of the FC at higher frequencies depends on the tracer while the noise floor is influenced by the MPI. The lower end of the resolution (here 4–5 mm) depends on the size of the FoV and the pattern that exhibit the lower FC in the SF over the FoV. A much lower resolution is obtained for the tracers La3, SPI, BNF with 4.59 mm, 4.56 mm, and 4.53 mm, respectively. It should be emphasized that these tracers have not been developed as MPI tracer systems. The obtained values from MPS were then correlated with the MPI resolution parameter *r* to estimate the prediction capability of the parameters for MPI performance (see Fig. 3). By linear regression (black lines) the correlation coefficients *R*^2^ = 0.65, 0.71, and 0.93 were determined for 
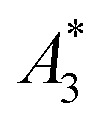
, *A*_5_/*A*_3_, and *k*_*A*>LOD_, respectively. Thus, the MPS parameter *k*_*A*>LOD_ enables a reliable prediction of achievable resolution *r* in MPI measurements, for the chosen MPS and MPI scanner setups parameters (see 2.3 and 2.4). Assuming a linear relation between *k*_*A*>LOD_ and *r* with *k*_*A*>LOD_ = 141(9) − 25(3) × *r* [in mm], *r* can be parametrized as

2 *r* = (141 − *k*_*A*>LOD_)/25

which according to Fig. 3 is applicable within the range *r* = 1 to 5 mm for the chosen gradient strength 2.5 T m^−1^ and two-voxel-analysis parameter. A different resolution limit would be resulting for other parameters in the two-voxel-analysis or for a different excitation field amplitude in the MPS measurements resulting in a different noise level to extract *k*_*A*>LOD_.

In addition, the size distribution (the values of *d*_hyd_ and PDI as obtained by DLS, see Table 1) was correlated to the resolution *r*. Only a poor correlation was found for the nine tracer systems (*R*^2^ = 0.04 for *d*_hyd_ and 0.001 for PDI). A precise relation between structure and size distribution of a tracer and the resulting resolution needs extensive and accurate characterization and should be further investigated. Very promising approaches for structural analyses are separation techniques that have already been described in ref. 12 and 31.

**Fig. 4:**
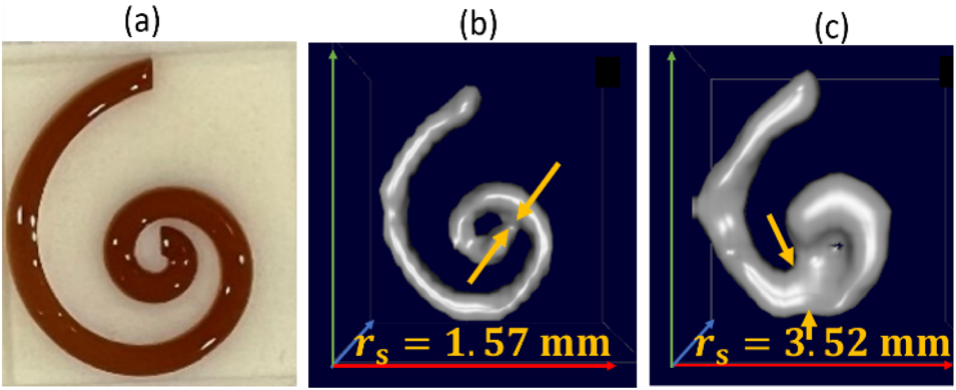
Photograph of a tracer-filled spiral phantom with 2 × 2 mm^2^ channel (a), reconstructed MPI image of the spiral phantom for SYN (b) with a “best” resolution of *r*_s_ = 1.57 mm marked by the yellow arrows, and (c) for SHP with a resolution *r*_s_ = 3.52 mm.

To demonstrate that we can predict the tracer MPI resolution of a tracer from the MPS parameter *k*_*A*>LOD_ we performed spiral phantom measurements (with a complete SF acquisition) for the tracer systems SYN and SHP, from which the resolution *r* for the MPI application was determined according to ref. 21, see Fig. 4.

The resolution of the spiral phantom *r*_s_ can be estimated in the image reconstruction from the minimum distance above which neighboring segments of the spiral channel are melding with each other so that the inner part (and the gap) of the spiral is no longer visible. From the MPI image reconstruction of the spiral a resolution *r*_s_ = 1.57 mm for SYN and *r*_s_ = 3.52 mm for SHP phantom was extracted. This is in very good agreement with *r* = 1.87 mm for SYN and *r* = 3.92 mm for SHP.

## 4. Conclusions

In this work, we evaluated nine different tracers by MPS to estimate their MPI capability and investigated the correlation of three MPS parameters (
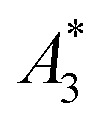
, *A*_5_/*A*_3_, and *k*_*A*>LOD_) and the hydrodynamic size distribution with the achievable MPI resolution *r* determined by the two-voxel-analysis. We found a weak correlation between the hydrodynamic size and the MPS parameters 
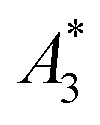
 and *A*_5_/*A*_3_ with the MPI resolution *r*. But based on our measurements, we conclude that *k*_*A*>LOD_ is a reliable parameter which enables the straightforward prediction of achievable MPI resolution *r* from MPS measurements – and this without the need of MPI phantom measurements. This was confirmed by MPI spiral phantom measurements with full SF acquisition.

## Conflicts of interest

The authors declare no conflict of interest.

## Supplementary Material
